# Hospital Laundries

**Published:** 1899-08-19

**Authors:** Conrad W. Thies

**Affiliations:** Secretary of the Royal Free Hospital, London, W.C.


### The Institutional Workshop.

### HOSPITAL LAUNDRIES.

By Conrad W. Thies, Secretary of the Royal Free Hospital, London, W.C.

One of the most difficult problems in connection with hospital management is that of the best method of dealing with the washing. The cost of this item of expenditure is necessarily large, and during the past few years has tended to greatly increase, owing to the greater degree of cleanliness which is demanded by the requirements of modern medical and surgical science in respect to everything that appertains to the patients and their treatment. The question which is most commonly asked by hospital managers who have this subject under consideration is whether it is advisable and economical for a hospital to undertake its washing in its own laundry or to send it out. The answer to this inquiry must, of course, depend mainly upon the special circumstances of each institution—its size, available space, and general appointments. In hospitals situated in towns, where the exigencies of space render it impracticable to provide a suitable site apart from the main buildings, I should certainly not recommend the establishment of a laundry in the basement beneath the wards, as is the case in some London hospitals; the serious objections to such an arrangement are too evident to require particularising.

As a general principle, I have no hesitation in expressing my opinion that every hospital ought, as far as is practicable, to be self-contained, and certainly ought to do its own washing. Apart from other considerations it is not in the public interest that hospital dirty linen, &c., should be sent away to outside laundries, where it must incur the risk of either giving or receiving infection. On the other hand, it is most desirable that the washing should be carried out under the direct control of the hospital officers.

It is difficult to make any reliable statement as regards the important consideration of economy. Some years since, when my committee were discussing the question of building a laundry, I made careful inquiries, and collected statistics on this point. I found, however, that it was practically impossible to ascertain with any degree of accuracy what was the actual cost of the washing at those London hospitals which had their own laundries. This arose from the fact that such important items as coal, water, gas, engineer's wages, repairs, &c., were included under their respective headings in the expenditure accounts, and it was not possible to apportion the actual share of cost of these items for which the laundry was responsible as apart from the general requirements of the hospital. As a result of the information obtained, my committee were convinced that some economy would be effected by having our own laundry, especially when the wear and tear of the linen were taken into consideration.

In order to render this article of practical utility to those who are interested in this question I will give some detailed particulars in reference to the laundry which was erected at the Royal Free Hospital five years since. The hospital has in residence a daily average of 140 to 143 patients, 50 nurses, seven resident officers, and 11 female servants. The washing averages about 4,200 pieces weekly. The laundry buildings occupy a site, 90 feet by 45 feet, in the rear of the hospital, and comprise a washhouse, drying room (with 14 drying horses), ironing and sorting room, and engine house; the cost, including machinery, was about £3,000. The washing appliances consist of an 8 h.p. steam engine, which drives two large rotary washing machines, two 32-inch hydro extractors, and a mangle. There are also provided various washing troughs, rinsing and boiling tanks, together with ironing stove and other necessary fixtures.

It is of the utmost importance that the machinery shall be adapted to the amount and character of the work to be performed. For instance, it is wasteful to have machines that are too large. I strongly advise that a laundry expert be consulted in the preparation of the specification for any new laundry.

The laundry staff consists of a forewoman and four women as ironers, &c., with one man to attend the washing machines. The engineer has charge of the engine and laundry machinery, and I estimate that this work occupies about one-third of his time. The wages of laundry hands vary very much in each locality; in London at present first-class hands command from £25 to £35 per annum, with board and lodging; ordinary ironers will receive 10s. per week, with meals during the day. It is an advantage to employ resident hands where it is possible to accommodate them, as better discipline is maintained; but, on the other hand, many good workers decline to accept employment except where they can sleep out. To secure the services of a capable, steady man to take charge of the washing machines it is necessary to pay from 25s. to 30s. per week. The following summarises the weekly cost of washing 4,200 pieces: Wages of six laundry hands, with cost of board and lodging, say, £6 10s.; part cost of engineer's and stoker's wages, £1; soap, soda, water, gas, &c., £2 10s.; total weekly cost, £10, or £520 per annum. The above is exclusive of the cost of providing steam, and the general repairs to machinery, &c., which may be estimated at £180, raising the total annual cost of the laundry to about £700, whereas the washing when sent out cost from £750 to £780 per annum for a somewhat smaller quantity.

Under the best of management it is very difficult to give entire satisfaction to all concerned in respect to their washing, and the greatest difficulty arises in reference to the personal washing for the nurses. This difficulty is often enhanced by the elaborate character of the caps, &c., which are worn at some institutions. It is customary to limit the number of articles which each nurse may send to the laundry.

The establishment of a laundry must necessarily involve a considerable addition to the work of those officers who are responsible for its management, but I do not think that any hospital which has once undertaken its own washing is likely to revert to the alternative of sending it out, unless compelled to do so by special circumstances.

